# Haemolytic-uremic syndrome due to infection with adenovirus: A case report and literature review

**DOI:** 10.1097/MD.0000000000009895

**Published:** 2018-02-16

**Authors:** Victoria Birlutiu, Rares Mircea Birlutiu

**Affiliations:** a Lucian Blaga University of Sibiu, Faculty of Medicine Sibiu; Academic Emergency Hospital Sibiu—Infectious Diseases Clinic; b Lucian Blaga University of Sibiu, Faculty of Medicine Sibiu, Romania.

**Keywords:** adenovirus infection, case report, hemolytic-uremic syndrome, non-STEC etiology

## Abstract

**Rationale:**

Haemolytic-uremic syndrome is a rare but serious complication of bacterial and viral infections, which is characterized by the triad of: acute renal failure, microangiopathic haemolytic anemia and thrombocytopenia, sometimes severe, requiring peritoneal dialysis. In Europe, hemolytic-uremic syndrome (HUS) in paediatric pathology is primarily caused by Shiga toxin-producing *Escherichia coli* (STEC) O157, followed by O26. Beside these etiologies, there are other bacterial and viral infections, and also noninfectious ones that have been associated to lead to HUS as well: in the progression of neoplasia, medication-related, post-transplantation, during pregnancy or associated with the antiphospholipid syndrome, systemic lupus erythematosus or family causes with autosomal dominant or recessive inheritance. In terms of pathogenesis, HUS is the result of endothelial injury, most commonly being a result of the action of Shiga toxin. The unfavorable prognosis factors being represented by the age of more than 5 years old, different etiologies from STEC, persistent oligoanuria, central nervous system and glomerular impairment, the association of fever with leukocytosis. HUS is responsible for 7% of cases of hypertension in infants, and an important cause of significant kidney damage in adults.

**Patient concerns:**

We present one case of HUS caused by adenovirus in a boy of 1 year and 7 months old with severe evolution, which required peritoneal dialysis.

**Diagnose:**

Stool sample repeated examination for adenovirus antigen was positive in 2 samples.

**Intervention:**

During hospitalization, the patient required 8 peritoneal dialysis sessions.

**Outcome:**

The renal function was corrected on discharge, the patient required cardiovascular monitoring 1 month after discharge.

**Lesson:**

Although the most common cause that leads to HUS remains STEC, other etiologies like viral ones that may be responsible for severe enteric infection with progression into HUS should not be neglected.

## 1 Introduction

Haemolytic-uremic syndrome (HUS) is a rare but serious complication to bacterial and viral infections, which is characterized by the triad of: acute renal failure, microangiopathic haemolytic anemia, and thrombocytopenia. From the pathophysiological point of view, HUS is a microangiopathy with predominantly renal and central nervous system impairment, associated with hemolytic anemia and acute renal failure,^[1–4]^ sometimes severe, requiring peritoneal dialysis. Although the most common cause of HUS in children remains Shiga toxin-producing *Escherichia coli* (STEC), there are other bacterial or viral etiologies that could lead to HUS. In Romania, during the first 4 months of 2016, there were 25 cases of infection caused by Shiga toxin-producing *E coli* O26-STEC,^[5]^ of which 19 developed HUS, patients aged between 5 and 38 months, resulting in 3 deaths. The cases were most likely attributable to the consumption of milk and cheese from a local producer, but other sources were not excluded as well. In the last 6 years, HUS was responsible for 101 cases, the highest prevalence was found in 2015 (25 cases) without any disease seasonality until this year; in February 2016, there were 12 cases of HUS, 2.5 times more cases than in the previous years. Although we focused on HUS risk associated with STEC infections in the territory, we diagnosed 1 case of HUS caused by adenovirus in a boy of 1 year and 7 months old, whose case we present below.

## 2 Case report

We present the case of a male Caucasian child, aged 1 year and 7 months, who was hospitalized in June 2016, after 7 days from the onset of fever (40°C), accompanied by diarrhea with mucus and blood, vomiting, impaired general condition, unresponsive to home-administered medication—ibuprofen, paracetamol, and diosmectite. At the time of admission, on physical examination, the following changes were noticed: pale skin, dry lips, pharyngeal hyperemia, respiratory rate of 36 breaths per minute, heart rate (HR) of 136 beats per minute (bpm), food refusal, fetid stools with mucus and blood.

Laboratory investigations revealed the following alterations: leukocytosis 33.090 × 10^9^ L, hemoglobin 11.8 g/L, hematocrit 33%. Normal renal and hepatic tests on admission. In progression: persistent leukocytosis (26.430 × 10^9^/L^3^) with neutrophilia 16.370 × 10^9^/L, increasing anemia 9.1–8.1 g/L with reticulocytosis (7.43%, 218.4 ×10^3^/μL, references values=3–120), lactate dehydrogenase 3060 U/L (references value = 615 U/L), changes suggestive for renal failure—blood urea nitrogen 69.7 to 177 mg/dL, creatinine 3.10 to 3.72 mg/dL, hepatocytolysis, gamma-glutamyl transferase (γGT) 126 U/L (references value 0–39 U/L), and a C-reactive protein of 49 mg/L (references value 0–10 mg/L). Peripheral blood smear revealed anisocytosis, poikilocytosis, and red blood cell fragmentation (schizocytes and rare microspherocytes). Bacteriological examinations of feces were negative for *Salmonella, Shigella, Campylobacter*, *Yersinia*, *E coli* 0157, enterohemorrhagic *E coli*; virulence molecular markers for verocytotoxin-producing *E coli* (VTEC/vtx1, vtx2), enteropathogenic *E coli* (EPEC/eae), enterotoxigenic *E coli* (ETEC elt, est) and enteroinvasive *E coli* (EIEC/ipaH) were negative. Repeated examination of stool sample for adenovirus, rotavirus, and norovirus were performed. Adenovirus antigen was positive in 2 samples. Due to the summer-fall seasonality of enterovirus infections in temperate climates which have been well established the stools were not test for enterovirus infection.

Other examinations imposed by the unfavorable progression: simple abdominal radiography revealed hydroaeric level in the left flank and gastric distension. Abdominal ultrasound emphasized renal cortical hyperecogenity, and intersplenorenal fluid lamina in the right iliac fossa, too. Cardiac ultrasound showed concentric left ventricular hypertrophy, with posterior hyperechoic pericardium, and secondary hypertension (renal parenchymal).

*During hospitalization*: persistent fever for the first 3 days, multiple diarrheal stools with mucus and blood, and food refusal. For proper monitoring of urine output, catheterization was performed, noting oligoanuria with a volume of 15 mL urine/24 hours under treatment with mannitol and furosemide. Biologically, there was noticed an increased nitrogen retention, and anemia associated with thrombocytopenia. Lack of response to therapy of renal insufficiency, occurrence of eyelid edema, high blood pressure (130/70 mm Hg), and sleepiness, imposed referral to a specialized nephrology clinic, where 8 peritoneal dialysis sessions were performed. Treatment of hypertension was associated with furosemide, metoprolol 15 mg/day, amlodipine 1.5 mg/day. The patient required cardiovascular monitoring 1 month after discharge. The renal function was corrected on discharge.

## 3 Discussions

In Europe, HUS in pediatric pathology is primarily produced by STEC O157, followed by O26. Since 2010, no fewer than 19 European countries reported 2350 cases produced by STEC O26 until 2014,^[6,7]^ with a peak in 2015, respectively, 463 cases. In the United States, the incidence of HUS in children under 6 years is of 6.1/100,000 population/year. In 2012, 274 cases related to diarrhea were reported.^[8]^ Other possible etiologies of HUS are: other bacterial infections (*Shigella dysenteriae*, *Salmonella*, *Campylobacter*, *Streptococcus pneumoniae, Mycoplasma, Legionella,* etc.), viral infections (enteroviruses, adenoviruses, *HIV*, *Epstein Barr* virus, herpes simplex, *Portillo* virus, etc.), but there are noninfectious causes as well: in the progression of neoplasia (pancreatic, gastric, and prostate cancer), medication-related (quinine, anticancer medication, antiplatelet drugs, contraceptives), post-transplantation (kidney, bone marrow, etc.) during pregnancy^[9]^ or associated with the antiphospholipid syndrome, systemic lupus erythematosus or family causes with autosomal dominant or recessive inheritance. In terms of pathogenesis, HUS is the result of endothelial injury, most commonly being a result of the action of Shiga toxin that attaches to the glycosphingolipid Gb3 globotriaosylceramide receptor,^[10,11]^ and other infectious or noninfectious triggers, with the release of vasoactive substances, glomerular microangiopathy and of small renal arterioles.^[12]^ Acute renal failure in the progression of HUS is reversible in 85% of cases under supportive care. The risk factors for the progression into HUS are the age of more the 5 years old, different etiologies from STEC, persistent oligoanuria, central nervous system, and glomerular impairment (80%). The combination of fever and leukocytosis represents an increased risk for HUS. Hemolytic uremic syndrome is responsible for 7% of cases of hypertension in infants, being the leading factor in chronic renal dysfunction in children, and an important cause of significant kidney damage in adults. This case is particular by the adenoviral etiology in a period dominated by *E coli* infections, with multiple loose stools that could have masked reduced diuresis and led to acute renal failure, associating other unfavorable prognostic factors like fever, leukocytosis, and non-STEC etiology. Monitoring of renal function, blood pressure, and heart rate will be needed in the next 3 to 5 years.

## 4 Informed consent

Written informed consent was obtained from the patient's parents for publication of this case report and any accompanying images. The study was accepted by the Ethics Committee of the hospital and they encourage publishing the article. A copy of the written consent is available for review by the editor-in-chief of this journal.

## 5 Conclusion

In February 2016, there was an increase of HUS cases, 2.5 times more cases than in the previous years. An unfavorable evolution is suggested by persistent fever, vomiting, or diarrhea associated with a water–electrolyte imbalance, the occurrence of neurologic manifestations and especially by oligoanuria which is difficult to be monitored in infants and young children. Early initiation of peritoneal dialysis remains the only means to improve the survival rate and the prognostic. Although the most common etiology of HUS remains STEC, other etiologies like viral etiologies should not be neglected, keeping in mind the fact that they might be responsible for severe enteric infection with progression into HUS.

## 6 Authors’ contributions

VB and RMB both contributed equally to this manuscript in terms of acquisition, analysis, and interpretation of data, conception and design, drafting the manuscript. Both authors read and approved the final manuscript.
